# Boltzmann–Loschmidt Dispute Reloaded: Quantum 150 Years Later

**DOI:** 10.3390/e28060594

**Published:** 2026-05-26

**Authors:** Leonardo Ermann, Alexei D. Chepelianskii, Dima L. Shepelyansky

**Affiliations:** 1 Departamento de Física Teórica, GIyA, Comisión Nacional de Energía Atómica, Av. del Libertador 8250, Buenos Aires 1429, Argentina; 2 LPS, Université Paris-Sud, CNRS, UMR 8502, F-91405 Orsay, France; 3 Laboratoire de Physique Théorique, Université de Toulouse, 31062 Toulouse, France

**Keywords:** time reversal, chaos, quantum chaos, cold atoms

## Abstract

The Boltzmann–Loschmidt dispute of 1876 questioned the possibility of a statistical irreversible description by time-reversible classical equations of motion of atoms. Here we show analytically and numerically that the quantum chaos diffusion of cold atoms, or ions, in a harmonic trap and pulsed optical lattice can be inverted back in time with up to 100% efficiency. This is in sharp contrast to classical evolution, where exponentially small errors break time reversibility. We argue that the existing experimental skills allow highlighting the Boltzmann–Loschmidt dispute from a quantum perspective.

## 1. Introduction

In 1872 Boltzmann formulated the statistical theory of entropy growth and thermalization based on the dynamical laws of classical motion of atoms [1]. A few years later in 1876, 150 years ago, this theory was objected to by Loschmidt [2], who pointed out that the dynamics of atoms is reversible in time, thereby raising the question of how an irreversible thermalization can appear from the reversible dynamical equations of motion. The reply of Boltzmann followed in 1877 [3]. The legend holds that, on the direct question of what happens with entropy and thermalization, if one inverts velocities of all atoms, he replied, *then go and invert them* [4]. The modern resolution of this Boltzmann–Loschmidt dispute is given by the theory of dynamical chaos in generic non-linear systems with positive Lyapunov exponent Λ and Kolmogorov–Sinai entropy $h_{KS}$, leading to an exponential instability of motion  [5,6,7,8]. This instability generates exponential growth in errors, thus breaking time reversibility even if time-reversal errors are negligibly small (see, e.g., [9]). It also leads to mixing with time at exponentially smaller scales in the phase space.

We should point out that the time reversibility problem of statistical laws, originated by the Boltzmann–Loschmidt dispute  [1,2], is still actively discussed by the scientific community in physics and philosophy (see, e.g., [10,11,12,13,14,15,16]).

However, the above discussions mainly occur in the frame of classical mechanics, while the reality is quantum. Quantum evolution is generally described by the linear Schrödinger equation, and chaotic mixing in a phase space stops at the Planck constant *ħ*, being protected by the Heisenberg uncertainty relation. Indeed, due to exponential divergence of classical trajectories, the Ehrenfest theorem for wave packet spreading [17] remains valid only for a logarithmically short Ehrenfest time $t_{E}∼\left|\ln\hbar \right|/\Lambda$, so, after $t_{E}$, the wave packet spreads exponentially over the main part of phase space  [18,19,20], and there is no instability for times $t>t_{E}$ [21,22]. Thus the time reversibility is preserved for the quantum evolution (see an example in [9]). The properties of time reversibility in systems of quantum chaos [20] have been studied in detail, being known as Loschmidt echo and fidelity decay (see, e.g., [23,24,25,26,27,28] and the Refs. therein).

The first experiments on time reversal were conducted with spin echos [29,30,31]. Later, time reversal was realized with acoustic and electromagnetic waves. This led to important and useful applications, including seismic analysis in geophysics (see, e.g., [32,33,34]). However, in far 1876, Boltzmann and Loschmidt discussed the time reversibility of atoms, and, for this system, an experimental realization of time reversal of atomic matter waves is rather nontrivial. A possible realization of time reversal of a quantum chaos evolution of cold atoms in a kicked optical lattice was proposed in [35] and for a Bose–Einstein condensate (BEC) of atoms in [36]. The main elements of this proposal are based on the possibility to transfer amplitude K/ħ of kicks from positive to negative values and the property of free propagation of atoms between kicks with a phase factor $U∼\exp(-iT{\left(n+\alpha \right)}^{2})/2)$, where a parameter *T* is proportional to a period between kicks and a fractional part α of momentum of atom p=n+α is not affected by kicks due to periodicity of the optical lattice. Therefore, a time reversal is realized by changing T=4π+ε to T=4π−ε and by K/ħ→−K/ħ at the middle of the time interval between kicks. However, this time reversal is exact only for atoms with a fractional quasimomentum α=0, while, for α close to zero, the time reversal degrades with an increase in time interval $t_{r}$, at which point the time-reversal operation is performed. Thus time reversal works only for a relatively small group of atoms with α≈0. The interactions between atoms also lead to a decrease in return signal [36]. The time reversal of atomic matter waves was realized in BEC experiments [37,38]. However, due to an approximate nature of time reversal for atoms with α≈0, only small values of time interval $t_{r}=5$ were realized with five kicks of forward and backward propagation.

## 2. Model Description

In this work we show that time reversal can be realized with cold atoms placed in a harmonic trap and kicked optical lattice and that in this system of quantum chaos almost all atoms return to the origin after a long reversal time $t_{r}$ with probability close to 100%. The system is described by the Hamiltonian

(1) $$\hat{H}=\left({\hat{p}}^{2}+{\omega_{0}}^{2}{\hat{x}}^{2}\right)/2+K\cos\left(q\hat{x}\right)\sum_{m}\delta \left(t-mT\right)\;,$$

where $\omega_{0}$ is a frequency of harmonic trap, momentum $\hat{p}$ and position $\hat{x}$ operators have the usual commutator $[\hat{p},\hat{x}]=-i\hbar$, and *T* is a time interval between kicks of optical lattice with a potential V(x)=Kcos(qx) with space period 2π/q. The classical dynamics of this system is described by the Hamiltonian equations, while the quantum evolution is described by the Schrödinger equation with the Planck constant *ħ*. Between kicks we have evolution in a harmonic potential, and a kick transfers a wave function ψ to $\bar{\psi }$ as $\bar{\psi }=\exp\left[-i\left(K/\hbar \right)\cos\left(qx\right)\right]\psi$. In our studies we use dimensionless units with atom mass and frequency, $\omega_{0}$ being unity, K,ħ,T being dimensionless and q=1 (the case of q≠1 is reduced to q=1 by a rescaling $K/\hbar \to K/\hbar_{eff}=K/\left(\hbar q^{2}\right)$.

The classical system (1) was introduced and studied in [39,40,41], known as Zaslavsky web map. This simple symplectic map describes a change in p,x variables after one period of time *T*. The dynamics depends on the ratio of oscillator period to the time between kicks R=2π/T. For R=3,4,6 the separatrix web covers the whole phase space plane (x,p) corresponding to the known result of a plane covered by triangles, squares and hexagons. The Kolmogorov–Arnold–Moser (KAM) theory [5,6,7,8] is not applicable for such a case, and even at small *K* values there are chaotic layers around separatrix lines of a width proportional to *K*. For large *K* values the whole phase space is chaotic without visible stability islands, and the system energy $E=<(p^{2}+x^{2})/2>$ is growing diffusely, with the number of kicks denoted as *t* in the following ($E\approx Dt,\;D\approx K^{2}/4$). The map on one period is $\bar{x}=p+K\sin x,\;\bar{p}=-x$, where bar marks new values of variables and R=4,q=1. A variety of images of classical dynamics in the phase space are available at [42].

The quantum evolution of system (1) is described by the quantum map for the wave function after one period of perturbation,

(2) $$\bar{\psi }=\exp\left(-i\left(\omega_{0}T\hat{n}+\varepsilon_{n}\right)\right)\exp\left[-i\left(K/\hbar \right)\cos q\hat{x}\right]\psi \;,$$

where $\hat{n}=\hat{a}+\hat{a}$ is the standard operator of oscillator quantum number *n*, and we assume that certain experimental imperfections at each map iteration induce random phases $-\varepsilon_{q}\leq \varepsilon_{n}\leq \varepsilon_{q}$ at oscillator levels; in the following we use $\omega_{0}=1,\hbar =q=1$. This quantum model at $\varepsilon_{q}=0$ was studied by different groups (see, e.g., [43,44,45,46,47,48] and Refs. therein). Quantum interference may lead to localization of classical diffusion, similar to a case of free cold atoms in a kicked optical lattice (see [18,19,49]), but there are also cases when the diffusion in energy is unlimited. Here we consider the case of R=4, with a duality between coordinate and momentum, when the system (1) can be reduced to the kicked Harper model with unlimited quantum diffusion (see, e.g., [43,50]). Numerically it is convenient to perform the quantum evolution (2) in the basis of oscillator eigenfunctions using the matrix elements of kick function between these eigenstates (see, e.g., [51] where the quantum evolution (1) was studied in presence of dissipation).

The time reversal of classical dynamics is done by inversion of velocities of all particles p→−p at the middle of free rotation between kicks. For the quantum evolution one cannot perform the complex conjugation $\psi \to \psi^{+}$ experimentally, but it is possible to invert evolution backwards in time by changing T=2π/R to $T^{\prime }=2\pi -T$ and replacing *K* amplitude with −K (at the moment of time reversal one should omit one kick replaced by $T^{\prime }$ rotation and then followed by kicks with −K amplitude and rotation periods $T^{\prime }$). This time-reversal operation works also for irrational *R* values. Also such a time reversal can be done if we add any integer number multiplied by 2π to *T* and $T^{\prime }$.

## 3. Time Reversal of Classical Chaos

**Figure 1:**
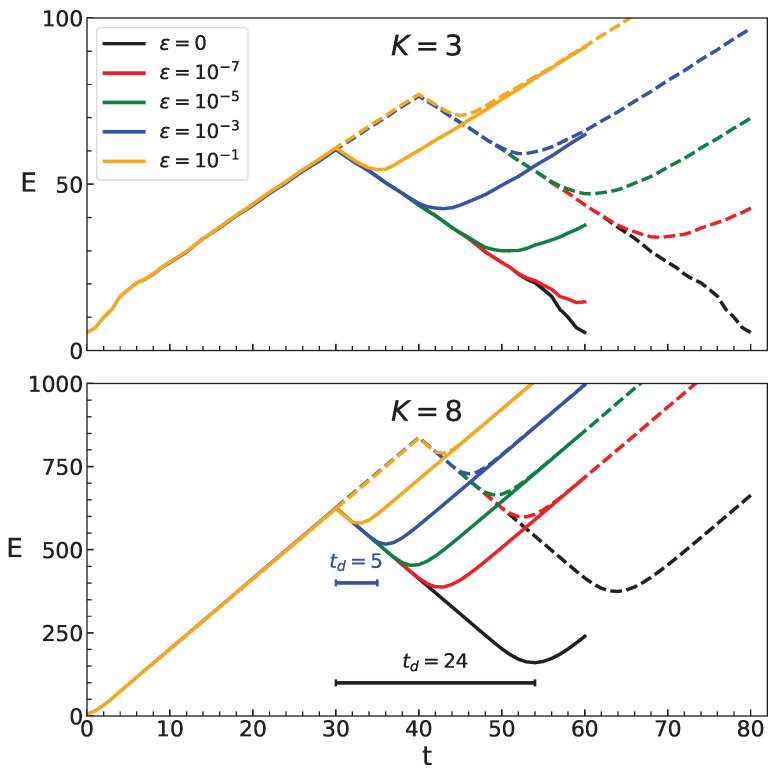
Time dependence of the mean energy E(t) for the classical system (1) at K=3 (**top panel**) and K=8 (**bottom panel**). Time reversal is performed at $t_{r}=30$ (solid curves) and $t_{r}=40$ (dashed curves) in the presence of noise with amplitude ε. Values of *E* are averaged over $10^{6}$ classical trajectories. At t=0, the initial distribution is a Gaussian centered at the unstable fixed point $\left(x_{0}=\pi ,p_{0}=0\right)$ with a standard deviation $\sigma =\sqrt{2}/2$. The recovery time $t_{d}$ is defined as the time during which the energy decays after time reversal, as illustrated in the bottom panel. For ε=0, no artificial noise is added, leaving only computer round-off errors at the double-precision level ($∼10^{-16}$). Here and in the below figures R=4.

The results for time reversal of classical dynamics of Zaslavsky web map with Hamiltonian (1) are shown in Figure 1 for energy time dependence E(t). Energy is averaged over $N=10^{6}$ trajectories with a Gaussian initial distribution centered at $x_{0}=\pi ,p_{0}=0$ and standard deviation $\sigma =\sqrt{2}/2$ in the phase space. Due to chaos there is a diffusive energy growth with time E=Dt at the diffusion rate $D\approx K^{2}/4$ corresponding to random phase approximation (actual values are $D/K^{2}\approx 0.16\;\left(K=3\right)$ and 0.33(K=8) due to presence of residual phase correlations, see [7,8]).

The time reversal is done at time moments $t_{r}=30$ and 40 at the middle between two kicks, as described above. For the case at K=3 the numerical simulations, done with double precision (round-off errors being about $\varepsilon ∼10^{-16}$), show the return to the initial state at time $t=2t_{r}$ for $t_{r}=30,40$ with the energy diffusion restarting for times $t>2t_{r}$. However, for K=8, similar to the Chirikov standard map [7], we have the Lyapunov exponent Λ≈ln(K/2)≈1.39, and the exponential error growth leads to a large accumulated round-off errors $10^{-16}\exp\left(\Lambda t_{r}\right)∼100$.

**Figure 2:**
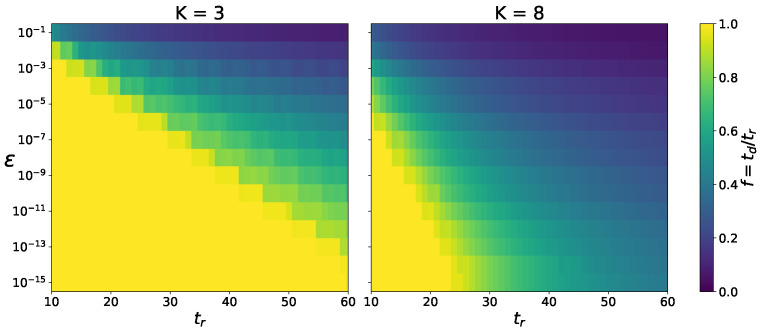
Dependence of the ratio of recovery and reversal times $f=t_{d}/t_{r}$ on noise amplitude ε and reversal time $t_{r}$, shown by color for K=3 (**left**) and K=8 (**right**). The values of *f* are averaged over $10^{6}$ trajectories for each color cell, where $t_{d}$ values are obtained as shown in Figure 1.

To illustrate the effect of errors we introduce after each time moment $0\leq t\leq 2t_{r}$ an additional random variation of momentum $p_{t+1}=p_{t}+\delta_{t}$ with $-\varepsilon \leq \delta_{t}\leq \varepsilon$. The effects of these artificial noise errors on energy anti-diffusion are shown in Figure 1. At a given ε this noise breaks time reversal, and the anti-diffusion back to the initial-state energy continues only during a finite time $t_{d}<t_{r}$. The dependence of the ratio $f=t_{d}/t_{r}$ on ε is shown in Figure 2 for different $t_{r}$ values. The results clearly show that the time scale $t_{d}$ is logarithmically short ($t_{d}\propto \left|\ln\varepsilon \right|/\Lambda$) due to exponential growth in errors. The time evolution of classical density distribution of trajectories is shown in video files of the Supplementary Materials (SupMat) for $t_{r}=30$ and K=3, ε=0.001.

## 4. Time Reversal of Quantum Chaos

**Figure 3:**
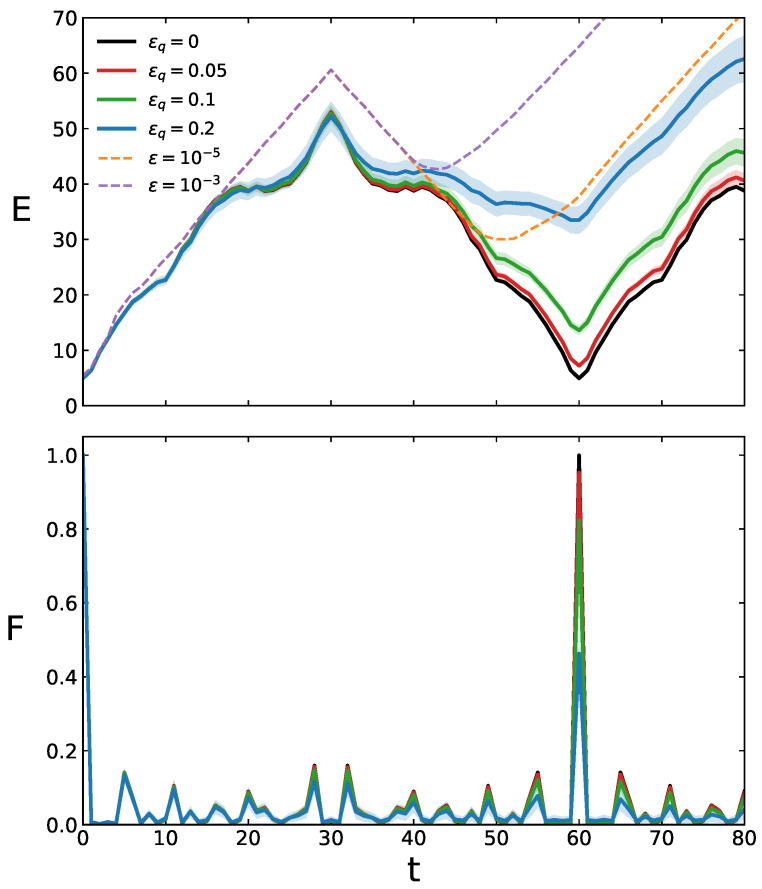
Time evolution of the mean energy E(t)=〈n〉 (**top panel**) and the quantum fidelity $F\left(t\right)=\;|\langle \psi \left(t=0,\varepsilon_{q}=0\right)|\psi \left(t,\varepsilon_{q}\right)\rangle {|}^{2}$ (**bottom panel**) for K=3, with ħ=q=1. Solid curves correspond to different quantum noise amplitudes $\varepsilon_{q}$: 0 (black), 0.05 (red), 0.1 (green), and 0.2 (blue). Shaded areas represent the standard deviation computed over 1000 noise realizations of the quantum map in Equation (2). Dashed curves show the classical mean energy E(t) averaged over $10^{6}$ trajectories for noise amplitude $\varepsilon =10^{-5}$ (orange) and $10^{-3}$ (purple). The initial classical and quantum state distributions, centered at $x_{0}=\pi ,p_{0}=0$, are shown in the top panels of Figure 4.

**Figure A1:**
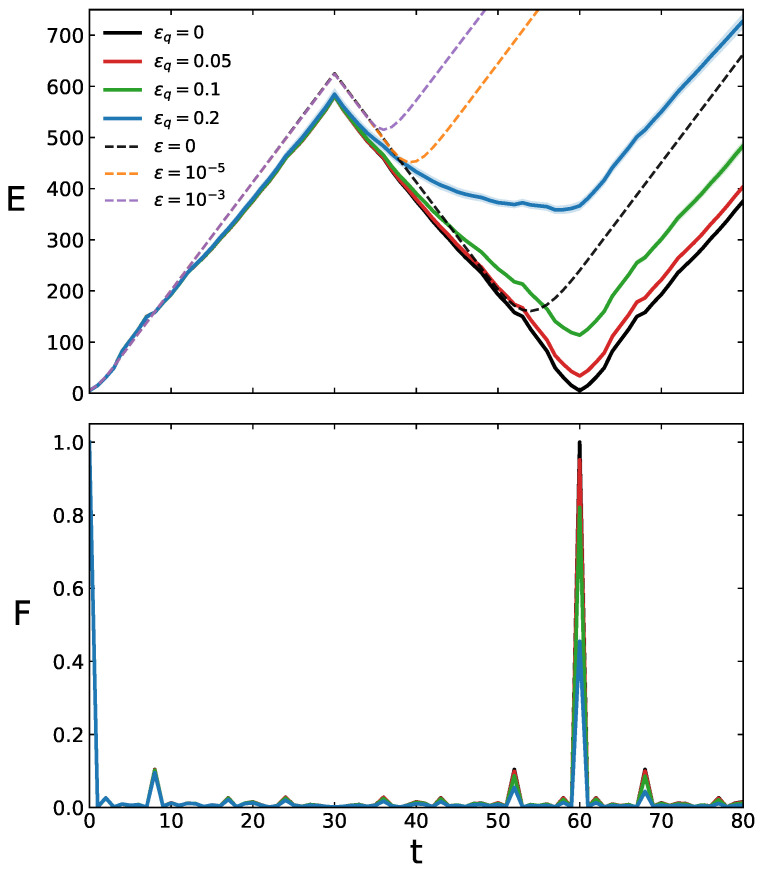
Time evolution of the mean energy E(t)=〈n〉 (**top panel**) and the quantum fidelity $F\left(t\right)=\;|\langle \psi \left(t=0,\varepsilon_{q}=0\right)|\psi \left(t,\varepsilon_{q}\right)\rangle {|}^{2}$ (**bottom panel**) for K=8, with ħ=q=1. Solid curves correspond to different quantum noise amplitudes $\varepsilon_{q}$: 0 (black), 0.05 (red), 0.1 (green), and 0.2 (blue). Shaded areas represent the standard deviation computed over 100 noise realizations of the quantum map in Equation (2). Dashed curves show the classical mean energy E(t) averaged over $10^{6}$ trajectories for noise amplitudes ε=0 (black), $10^{-5}$ (orange) and $10^{-3}$ (purple). The initial classical and quantum state distributions are centered at $x_{0}=\pi ,\;p_{0}=0$.

The time reversal of quantum chaos diffusion in (2) with $t_{r}=30$ and K=3 is shown in Figure 3 (top panel). For $t\leq t_{r}$ there is diffusive growth in oscillator energy E(t), being the same as for the classical case in Figure 1. After the time reversal there is anti-diffusion back to the initial state during $t_{r}<t\leq 2t_{r}$, and for $t>2t_{r}$ the quantum diffusion restarts again. For the quantum fidelity $F\left(t\right)=|\langle \psi \left(t=0,\varepsilon_{q}=0\right)|\psi \left(t,\varepsilon_{q}\right)\rangle {|}^{2}$ there is a decrease in *F* for $0<t\leq t_{r}$, followed by its revival back to $F(2t_{r})=1$ for $\varepsilon_{q}=0$. In the presence of quantum phase noise $\varepsilon_{n}$ the time-reversal signal is slowly decreasing with an increase in noise amplitude $\varepsilon_{q}$, as indicated in Figure 3. However, the quantum evolution remains much more stable with respect to quantum errors compared to the case of classical chaotic dynamics with classical errors. Similar results for K=8,ħ=1 are shown in Appendix A Figure A1.

**Figure 4:**
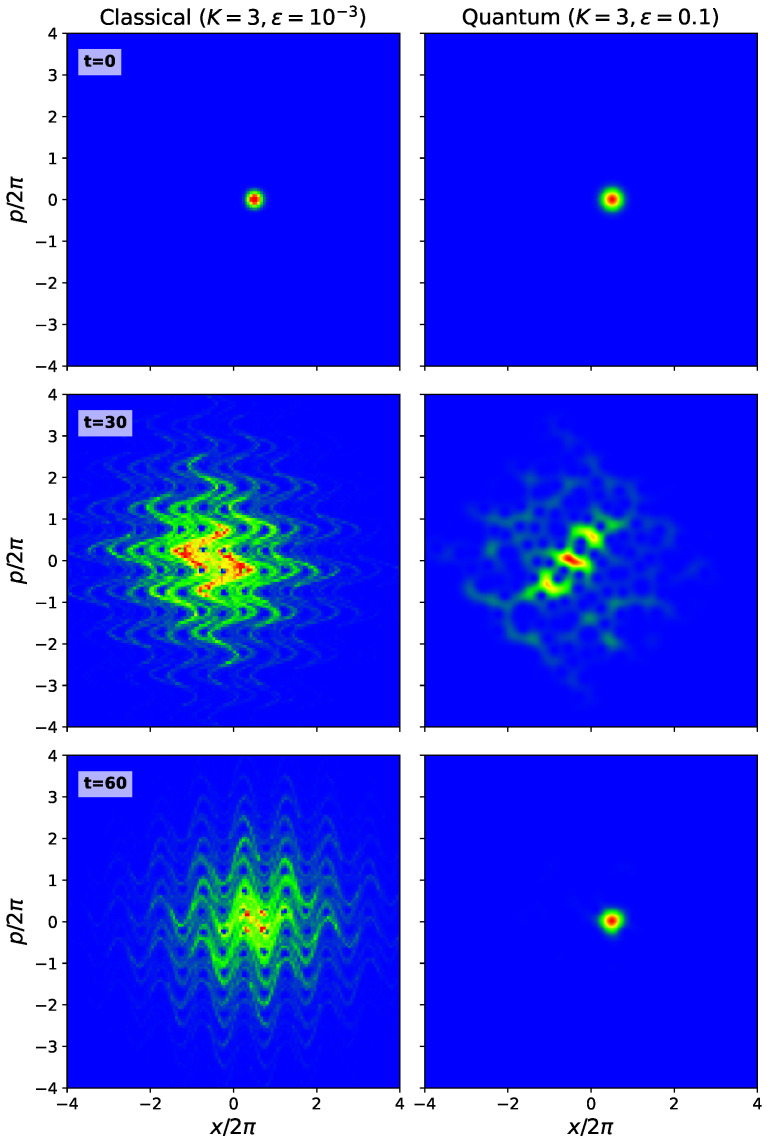
Phase-space comparison of the time-reversal dynamics for classical probability densities (**left column**) and quantum Husimi distributions [20,25] (**right column**). The rows, from top to bottom, correspond to the initial state at t=0, the state at the reversal time $t=t_{r}=30$, and the final state at $t=2t_{r}=60$. Classical distributions are computed from an ensemble of $10^{6}$ trajectories with a noise amplitude $\varepsilon =10^{-3}$. The quantum Husimi distributions are shown for one noise realization with amplitude $\varepsilon_{q}=0.1$. All initial states are centered at $\left(x_{0},p_{0}\right)=\left(\pi ,0\right)$. The system parameters are K=3 and ħ=1, with non-linearity exponent q=1; here red color is for maximal density, blue for zero.

The examples of classical and quantum distributions in the phase space (x,p) at time moments $t=0,t_{r},2t_{r}$ are shown in Figure 4. At $t=t_{r}=30$ the classical and quantum distributions cover a large area in the phase space. However, at the return time $t=2t_{r}=60$ the quantum distribution, with noise error amplitude $\varepsilon_{q}=0.1$, returns almost perfectly to the initial state (with fidelity $F(2t_{r})\approx 0.85$). In contrast, for the classical chaotic dynamics, with error amplitude ε=0.001, the time reversal is broken and a big fraction of trajectories continue to spread diffusely in the phase space. The videos of this classical and quantum evolution are presented in SupMat.

**Figure 5:**
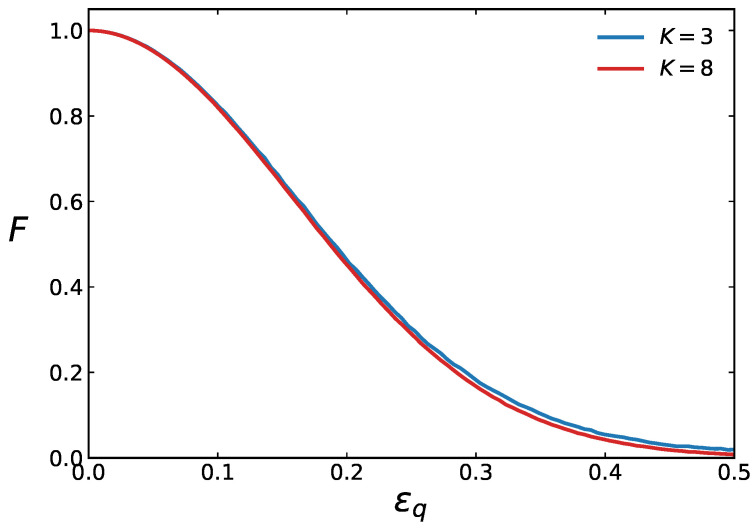
Dependence of the quantum fidelity $F(t=2t_{r})$ on the quantum noise amplitude $\varepsilon_{q}$ for K=3 (blue) and K=8 (red) at a fixed reversal time $t_{r}=30$. Values of *F* are averaged over 1000 quantum noise realizations.

**Figure 6:**
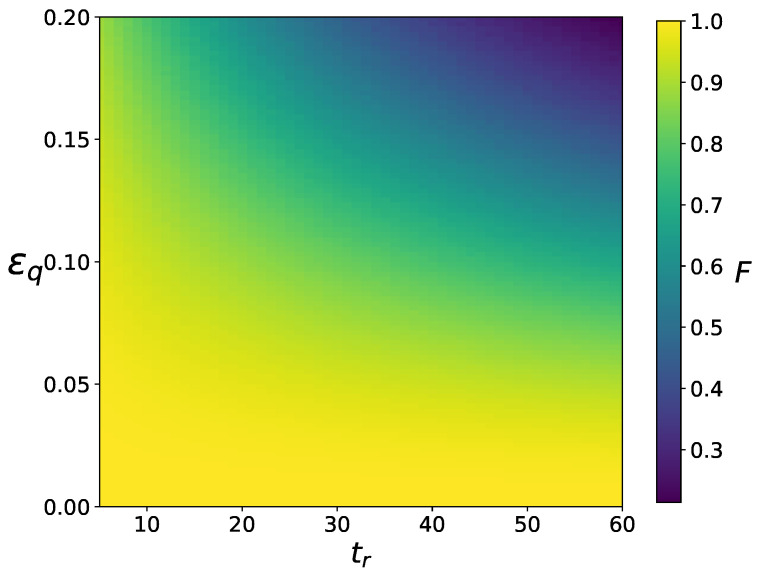
Dependence of the quantum fidelity $F(t=2t_{r})$ on the reversal time $t_{r}$ and the quantum noise amplitude $\varepsilon_{q}$ for K=3, with ħ=q=1. Values of *F*, represented in color scale, are averaged over 1000 quantum noise realizations; the dependence of classical fidelity is shown in Appendix A Figure A2.

In Figure 5 we show the dependence of fidelity $F(t=2t_{r}=60)$ on the quantum noise amplitude $\varepsilon_{q}$. It can be approximately described by the relation $F∼\exp(-G{\varepsilon_{q}}^{2}t_{r})$, where *G* depends on chaos parameter *K* and *ħ* in agreement with general properties of Loschmidt echo decay (see, e.g., [25,28] and Refs. therein). The results of Figure 6 also show that the time-reversal fidelity is very stable with respect to quantum errors, with a drastic difference in the exponential sensitivity of classical dynamics in terms of classical errors shown in Figure 2.

The comparison of energy time dependence E(t) for classical and quantum cases (top panels of Figure 1 and Figure 3) shows that, even at the strongest amplitude of quantum noise ε=0.2, there is still a visible decrease at $t=2t_{r}$ for quantum energy related to a fidelity peak at that time even at this relatively strong noise. For the classical case the exponential instability is much stronger and there is no dip for $E(t=2t_{r})$ even for a noise amplitude ε=0.001. Let us note that, for the acoustic time reversal done by the Fink group [32] on a small portion of a chaotic billiard perimeter, it was sufficient to observe a strong time-reversal peak.

We note that the described time-reversal procedure works for noninteracting particles, while their interactions break reversibility, which enables studying effects of interactions. However, as shown in Figure 1 and Figure 2, the origin of a breaking of statistical description (e.g., diffusion) from time-reversal dynamical equations of motion is the emergence of dynamical chaos with its exponential instability for noninteracting particles. Thus, for the Lorentz gas (particle moving in a gas of fixed disks), it is mathematically proven that the evolution of an initial particle density converges to the solution of the Boltzmann equation [52].

It is known that, for quantum many-body systems, interactions can lead to a relaxation of an initial state to a thermal distribution (see, e.g., [53]). In such a quantum system certain signs of Lyapunov instability can be present (see, e.g., [54]) but only for a short Ehrenfest time [18]. The time reversal for quantum many-body systems with a time-symmetric Hamiltonian can be done by a phase conjugation $\psi \to \psi^{*}$ at symmetric moments of time [55]. Here we describe a procedure when the phase conjugation and time reversal are done for noninteracting cold atoms that have chaotic classical dynamics. The interactions between atoms can be switched off via Feshbach resonance, as demonstrated experimentally in [38]. Also, with the help of Feshbach resonance and a change in magnetic field, a scattering length of atoms can be changed from a positive to an opposite negative value. This enables performing a phase conjugation of the interacting part of the Hamiltonian of cold atoms that, in combination with the procedure described above, in principle makes it possible to perform time reversal of quantum evolution for a complete many-body quantum interacting system.

In the case of a hybrid system where some degrees of freedom are described by the classical dynamics and other ones by the quantum equations (see, e.g., [56]), a chaotic dynamics of classical degrees may lead to exponential instability and time-reversal breaking in the whole hybrid system.

## 5. Possible Experiments

For cold atoms in a kicked optical lattice a time interval between kicks was about T∼1 μs–30 μs [49,57]. The kick period can be even longer, being comparable with a trap oscillator period of cold atoms with long coherence times. As discussed in [45] the quantum Zaslavsky web map can also be realized with cold ion traps, with oscillation frequencies of about 1 MHz [58]. The kick amplitude in the experiments [37,38,49,57] was typically K/ħ ∼ 2–3, and, at present, with a stronger optical lattice laser power, it can be increased by a factor of two or three, corresponding to the dimensionless values presented in this work.

We expect that, in real experimental conditions, various sources of noise and decoherence will be present. Therefore, e.g., a residual scattering of atoms on each other and other effects of finite temperature can produce certain phase noise for quantum evolution. The presented results show that the quantum time reversal is rather robust with respect to quantum noise, which supports the expectation that the time-reversal signal will also be robust in real experiments.

## 6. Discussion

We highlighted the Boltzmann–Loschmidt dispute on the time reversibility of statistical laws [2,3] emerging from dynamical equations of motion from the modern view of quantum mechanics of cold atoms, or ions, in a harmonic trap and pulsed optical lattice. We argue that the actual experimental abilities enable realizing time reversal of quantum chaos diffusion of cold atoms with almost 100% efficiency.

## Figures and Tables

**Figure A2:**
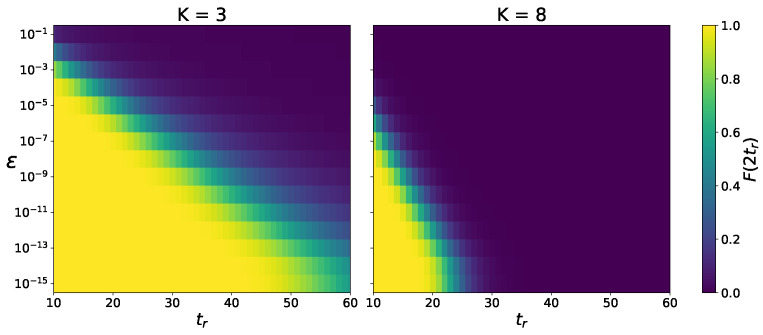
Dependence of the classical fidelity $F(2t_{r})$ at the return time $2t_{r}$ on the noise amplitude ε and reversal time $t_{r}$. Other parameters are the same as in Figure 2.

## Data Availability

The data presented in this study are available on request from the corresponding author due to quantity.

## Supplementary Materials

The following supporting information can be downloaded at: https://www.mdpi.com/article/10.3390/e28060594/s1, supmatvideo1.mp4, supmatvideo2.mp4.

## Appendix A

Appendix includes Figure A1 and Figure A2, and Supplementary Materials (SupMat) contain two video files in MP4 format.

For Figure A1, the system parameters are similar to those of Figure 3 in the main text.

SupMat video file supmatvideo1.mp4 shows the classical time evolution of the density distribution in the phase space (x,p) (bottom panel). The top panel shows the dependence of the classical energy E(t) and classical fidelity F(t) at a noise amplitude of $\varepsilon =10^{-3}$, obtained with $10^{6}$ classical trajectories for one noise realization and the parameters of Figure 4 (K=3,q=1). The classical fidelity F(t) is defined as an overlap between the initial classical phase-space distribution at t=0 and the distribution at time *t*,

(A1) $$F\left(t\right)=\frac{\int \rho (x,p,0)\rho (x,p,t)\;dxdp}{\sqrt{\int \rho^{2}\left(x,p,0\right)\;dxdp\int \rho^{2}\left(x,p,t\right)\;dxdp}},$$

where ρ denotes the classical coarse-grained density distributions.

SupMat video file supmatvideo2.mp4 shows the quantum time evolution of the Husimi density distribution in the phase space (x,p) (bottom panel) shown for one noise realization. The top panel shows the dependence of the quantum energy E(t) and quantum fidelity F(t) at a noise amplitude of $\varepsilon_{q}=0.1$ for one noise realization and the parameters of Figure 4 (K=3,ħ=1,q=1).

## References

[1] Boltzmann L. (1872). Weitere Studien über das Wärmegleichgewicht unter Gasmolekülen. Wien. Berichte.

[2] Loschmidt J. (1876). Über den Zustand des Wärmegleichgewichts eines Systems von Körpern mit Rücksicht auf die Schwerkraft. Sitzungsberichte Der Akad. Der Wiss. Wien.

[3] Boltzmann L. (1877). Über die Beziehung eines allgemeine mechanischen Satzes zum zweiten Haupsatze der Wärmetheorie. Sitzungsberichte Der Akad. Der Wiss. Wien.

[4] Mayer J.E., Goeppert-Mayer M. (1977). Statistical Mechanics.

[5] Arnold V., Avez A. (1968). Ergodic Problems of Classical Mechanics.

[6] Cornfeld I.P., Fomin S.V., Sinai Y.G. (1982). Ergodic Theory.

[7] Chirikov B.V. (1979). A universal instability of many-dimensional oscillator systems. Phys. Rep..

[8] Lichtenberg A., Lieberman M. (1992). Regular and Chaotic Dynamics.

[9] Shepelyansky D.L. (1983). Some statistical properties of simple classically stochastic quantum systems. Phys. D.

[10] Sklar L. (1993). Physics and Chance: Phylosophical Issues in the Foundations of Statistical Mechanics.

[11] Lindey D. (2001). Boltzman’s Atom: The Great Debate That Launched a Revolution in Physics.

[12] Bader A., Parker L. (2001). Joseph Loschmidt, Physicist and Chemist. Phys. Today.

[13] Price H. (2002). Boltzmann’s Time Bomb. Br. J. Philosiphy Sci..

[14] Uffink J. (2006). Compendium of the Foundations of Classical Statistical Physics. https://philsci-archive.pitt.edu/2691/1/UffinkFinal.pdf.

[15] Uffink J. (2024). Boltzmann’s Work in Statistical Physics. Stanford Encyclopedia of Philosophy Archive. https://plato.stanford.edu/archives/win2024/entries/statphys-Boltzmann/.

[16] Weidenmuller H.A. (2005). The rise of stochasticity in physics. Eur. Phys. J. Plus.

[17] Ehrenfest P. (1927). Bemerkung über die angenaherte Gultigkeit der klassischen Mechanik innerhalb der Quantenmechanik. Z. Fur Phys..

[18] Chirikov B.V., Izrailev F.M., Shepelyansky D.L. (1981). Dynamical stochasticity in classical and quantum mechanics. Sov. Sci. Rev. C.

[19] Chirikov B.V., Izrailev F.M., Shepelyansky D.L. (1988). Quantum chaos: Localization vs. ergodicity. Phys. D.

[20] Haake F. (2001). Quantum Signatures of Chaos.

[21] Shepelyanskii D.L. (1981). Dynamical stochasticity in nonlinear quantum systems. Theor. Math. Phys..

[22] Shepelyansky D. (2020). Ehrenfest time and chaos. Scholarpedia.

[23] Peres A. (1984). Stability of quantum motion in chaotic and regular systems. Phys. Rev. A.

[24] Jalabert R.A., Pastawski H.M. (2001). The semiclassical tool in complex physical systems: Mesoscopics and decoherence. Adv. Solid State Phys..

[25] Frahm K.M., Fleckinger R., Shepelyansky D.L. (2004). Quantum chaos and random matrix theory for fidelity decay in quantum computations with static imperfections. Eur. Phys. J. D.

[26] Gorin T., Prosen T., Seligman T.H., Znidaric M. (2006). Dynamics of Loschmidt echos and fielity decay. Phys. Rep..

[27] Jacquod P., Petitjean C. (2009). Decoherence, entanglement and irreversibility in quantum dynamical systems with few degrees of freedom. Adv. Phys..

[28] Gousev A., Jalabert R.A., Pastavsli H.M., Wisniacki D.A. (2012). Loschmidt echo. Scholarpedia.

[29] Hahn E.L. (1950). Spin echos. Phys. Rev..

[30] Bagguley D.M.S. (1992). Pulsed Magnetic Resonance: NMR, ESR, and Optics: A Recognition of E. L. Hahn.

[31] Pastawski H.M., Levstein P.R., Usaj G., Raya J., Hirschinger J. (2000). A nuclear magnetic resonance answer to the Boltzmann-Loschmidt controversy?. Phys. A.

[32] Fink M. (2001). Chaos and time-reversed acoustics. Phys. Scr..

[33] Lerosey G., de Rosny J., Tourin A., Derode A., Montaldo G., Fink M. (2004). Time Reversal of Electromagnetic Waves. Phys. Rev. Lett..

[34] Larmat C.S., Guyer R.A., Johnson P.A. (2010). Time-reversal methods in geophysics. Phys. Today.

[35] Martin J., Georgeot B., Shepelyansky D.L. (2008). Cooling by time reversal of atomic matter waves. Phys. Rev. Lett..

[36] Martin J., Georgeot B., Shepelyansky D.L. (2008). Time reversal of Bose-Einstein condensates. Phys. Rev. Lett..

[37] Ullah A., Hoogerland A.D. (2012). Experimental observation of Loschmidt time reversal of a quantum chaotic system. Phys. Rev. E.

[38] Cao A., Sajjad R., Mas H., Simmons E.Q., Tanlimco J.L., Nolasco-Martinez E., Shimasaki T., Kondakci H.E., Galitski V., Weld D. (2022). Interaction-driven breakdown of dynamical localization in a kicked quantum gas. Nat. Phys..

[39] Zaslavskii G.M., Zakharov M.Y., Sagdeev R.Z., Usikov D.A., Chernikov A.A. (1986). Stochastic web and diffusion of particles in magnetic field. Sov. Phys. JETP.

[40] Chernikov A.A., Sagdeev R.Z., Usikov D.A., Zakharov M.Y., Zaslavsky G.M. (1987). Minimal chaos and stochastic web. Nature.

[41] Zaslavsky G. (2007). Zaslavsky web map. Scholarpedia.

[42] The Zaslavsky Web Map Generator. https://www.russellcottrell.com/fractalsEtc/Zaslavsky.htm.

[43] Shepelyansky D., Sire C. (1992). Quantum evolution in a dynamical quasi-crystal. Eur. Lett..

[44] Dana I. (1994). Quantum suppression of diffusion on stochastic web. Phys. Rev. Lett..

[45] Gardiner S.A., Cirac J.I., Zoller P. (1997). Quantum chaos in an ion trap: The delta-kicked harmonic oscillator. Phys. Rev. Lett..

[46] Kells G.A., Twamley J., Heffernan D.M. (2004). Dynamical properties of the delta-kicked harmonic oscillator. Phys. Rev. E.

[47] Carvalho A.R.R., Buchleitner A. (2004). Web-assisted tunneling in the kicked harmonic oscillator. Phys. Rev. Lett..

[48] Billam T.P., Gardiner S.A. (2009). Quantum resonances in an atom-optical *δ*-kicked harmonic oscillator. Phys. Rev. A.

[49] Moore F.L., Robinson J.C., Bharucha C.F., Sundaram B., Raizen M.G. (1995). Atom optics realization of the quantum *δ*-kicked rotor. Phys. Rev. Lett..

[50] Artuso R. (2011). Kicked Harper model. Scholarpedia.

[51] Chepelianskii A.D., Shepelyansky D.L. (2026). Kicked fluxonium with quantum strange attractor. Physics.

[52] Boldrighini C., Bunimovich L.A., Sinai Y.G. (1983). On the Boltzmann Equation for the Lorentz Gas. J. Stat. Phys..

[53] Rigol M., Dunjko V., Olshanin M. (2008). Thermalization and its mechanism for generic isolated quantum systems. Nature.

[54] Maldacena J., Shenker S.H., Stanford D. (2016). A bound on chaos. J. High Energy Phys..

[55] Landau L.D., Lifshitz E.M. (1974). Quantum Mechanics.

[56] Singh A.K., Chotorlishvili L., Srivastava S., Tralle I., Toklikishvili Z., Berakdar J., Mishra S.K. (2020). Generation of coherence in an exactly solvable nonlinear nanomechanical system. Phys. Rev. B.

[57] Chabe J., Lemarie G., Gremaud B., Delande D., Sziftgiser P., Garreau J.C. (2008). Experimental Observation of the Anderson Metal-Insulator Transition with Atomic Matter Waves. Phys. Rev. Lett..

[58] Pogorelov I., Feldker T., Marciniak C.D., Postler L., Jacob G., Krieglsteiner O., Podlesnic V., Meth M., Negnevitsky V., Stadler M. (2021). Compact Ion-Trap Quantum Computing Demonstrator. PRX Quantum.

